# Predictors of narrow angle detection rate—a longitudinal study of Massachusetts residents over 1.7 million person years

**DOI:** 10.1038/s41433-020-1003-0

**Published:** 2020-06-03

**Authors:** Cecilia S. Lee, Michael L. Lee, Ryan T. Yanagihara, Aaron Y. Lee

**Affiliations:** 1 Department of Ophthalmology, University of Washington, Seattle, WA USA; 2 Department of General Internal Medicine, University of Washington, Seattle, WA USA

**Keywords:** Physical examination, Health occupations, Epidemiology

## Abstract

**Background/Objectives:**

To determine the predictors of narrow angle detection in a United States population-based cohort.

**Materials and methods:**

This was a retrospective cohort study using the Massachusetts All-Payer Claims Database. Demographic information of all patients and eye care provider information during the years 2011–2015 were extracted from Massachusetts All Payers Claims Data. All payers who received eye care during 1/1/2012–12/31/2015 without any previous eye visit during 2011 were included in the analyses. Laser peripheral iridotomy was identified by Current Procedural Terminology code 66761. Narrow angle detection was defined as the diagnosis of narrow angles by diagnosis code followed by a laser peripheral iridotomy procedure. Different predictors of narrow angle detection were evaluated using Kaplan–Meier curves with the log rank and Cox regression modeling.

**Results:**

A total of 1,082,144 patients were included. The hazard ratio of narrow angle detection increased with age compared to the reference group of 0–10 years: 21–30 years of age (hazard ratio = 4.5), 31–40 (10.5), 41–50 (27.9), 51–60 (46.1), 61–70 (68.4), 71–80 (56.8) (all *p* < 0.0002), was 1.47 times higher in women and 1.85 times higher if evaluated by ophthalmologists compared to optometrists, after controlling for provider × time interaction.

**Conclusion:**

Older age and female sex are associated with narrow angles. The rate of narrow angle detection was significantly higher if patients are seen by ophthalmologists compared to optometrists only. Evaluation with an ophthalmologist may be important for patients at high risks for developing primary angle closure glaucoma.

## Introduction

Primary angle closure glaucoma (PACG) is one of the leading causes of blindness worldwide, affecting ~26% of the glaucoma population globally [1]. Primary angle closure suspect is defined as occludable narrow angles or iridotrabecular contact on gonioscopy. If undetected, one in four primary angle closure suspect population will develop primary angle closure with elevated intraocular pressures and/or posterior synechiae in 5 years [2]. However, the larger, more recent Zhongshan Angle Closure Prevention (ZAP) Trial found that over 6 years, less than 1% of primary angle closure suspects progressed to PACG [3]. In those who develop disease, permanent optic nerve damage and irreversible blindness may occur. The prevalence of PACG varies from 0.1 to 10% with predilection to particular races such as Eskimos or East and Southeast Asians [4–8]. The prevalence of primary angle closure and occludable narrow angles are even more difficult to assess due to requiring a population screening program with reliable diagnostic methods [1, 9].

Key in prevention of PACG relies on timely detection of occludable narrow angles and referrals for laser peripheral iridotomy (LPI) if indicated [10]. The gold standard of diagnosing occludable narrow angles is dynamic gonioscopy, but this is not commonly performed by all eye care providers [11, 12]. Given that eye care delivery is often shared by optometrists and ophthalmologists in the USA, evaluating the quality of gonioscopy examination and detection of narrow angles by eye care providers is crucial to assessing PACG care and prevention.

A recent rise in Big Data due to electronic health records and medical claims databases allows novel approaches to epidemiology studies. They have the potential to provide large-scale resources for determining the prevalence and incidence estimates in populations in addition to a variety of risk factors. However, accurate measurements of new diseases (the numerators) and the number of person years at risk (the denominators) can be difficult to obtain from these databases [13].

Massachusetts (MA) All Payers Claims Database (APCD) by the Center for Health Information and Analysis provides a unique opportunity to assess the longitudinal medical history of every state resident and the care received from all providers (i.e., optometrists and ophthalmologists) in MA. Because APCD includes all payers regardless of insurance type, it allows a more accurate assessment of incidence rates than other large databases such as Medicare Claims or clinical registries. We sought to use this rich dataset to assess the incidence and risk factors of narrow angle diagnoses requiring LPI in the population of MA with particular interests in different provider types.

## Methods

### Data collection

The American Reinvestment and Recovery Act created the MA APCD program to enable detailed, comparative financial and health research. Longitudinal claims data paid for every state resident including all public and private payers are included in MA APCD. All payers claims data during the years 2011–2015 except from the Medicaid population (i.e., all Medicare, private insurance, and uninsured populations) were included in this study (version 5.0). The Institutional Review Board of the University of Washington, Seattle approved this study. The study was conducted in accordance with the Declaration of Helsinki.

### Patient cohort and provider data

The sociodemographic and clinical data from the patient cohort were extracted. The LPI procedure was identified by Current Procedural Terminology (CPT) code 66761. Narrow angles were defined as the diagnosis of narrow angles (either ICD-9 354.02 or ICD-10 H40.03*) followed by an LPI procedure.

Study inclusion criteria were any new patients being seen by an eye care provider from 1/1/2012 to 12/31/2015 without any previous eye care provider visit during 1/1/2011–12/31/2011. Patients were grouped as either initially seen by optometrists or initially seen by ophthalmologists. Patients were then followed forward in the available data to determine whether LPI was performed by determining whether CPT code 66761 was coded for any available CPT codes associated with an encounter. The patients were censored when the last known encounter with an eye care specialist occurred in the dataset.

Since optometrists do not perform LPIs in the state of MA, the event time for optometrist was defined as the first date of the encounter at which the optometrist diagnosed narrow angles which subsequently led to an LPI being performed within 90 days. If a patient was seen initially by an optometrist and then seen by an ophthalmologist, then the patient was censored on the last day that they were seen by an optometrist before being seen by an ophthalmologist. Conversely, if the patient was initially seen by an ophthalmologist and then seen by an optometrist, then the patient was censored on the last day that they were seen by an ophthalmologist before seeing the optometrist.

### Statistical analyses

Survival analyses were performed with Kaplan–Meier curves with log rank comparisons and Cox regression. Data manipulations and all analyses were performed with Python (python.org) and R (R project; http://www.R-project.org). All ages from the dataset that were coded as older than 75 were converted to 76.

## Results

A total of 3,761,397 patients were seen by eye care providers during 2011–2015. A total of 2,078,592 patients were seen by optometrists only, 1,024,606 by ophthalmologists only, and 658,199 by both optometrists and ophthalmologists. Mean age of patients seen by an eye care provider at first visit was 44.3 (SD: 20.2) and 1,609,685 (42.8%) were male.

A total of 1,082,144 patients met the inclusion criteria (i.e., new patients seen during 2012–2015). In our inception cohort, approximately 63.5% were seen by an optometrist initially and generally older patients were seen at first visit by ophthalmologists compared to optometrists (Table 1). Similar sex distributions were seen among the two provider types. The total number of person years captured in the analysis was 1,668,693 person years.

**Table 1:** Baseline demographic factors of study population.

	Initially seen by optometrist	Initially seen by ophthalmologist	Total
*n*	706,581	375,563	1,082,144
Age decade (*n*, %)^a^			
<10	29,062 (4.1)	28,744 (7.7)	57,806 (5.3)
10–19	79,964 (11.3)	25,293 (6.7)	105,257 (9.7)
20–29	88,591 (12.5)	20,224 (5.4)	108,815 (10.1)
30–39	86,626 (12.3)	24,126 (6.4)	110,752 (10.2)
40–49	128,102 (18.1)	47,038 (12.5)	175,140 (16.2)
50–59	141,941 (20.1)	72,090 (19.2)	214,031 (19.8)
60–69	117,669 (16.7)	106,585 (28.4)	224,254 (20.7)
70+	34,540 (4.9)	51,420 (13.7)	85,960 (7.9)
Sex (*n*, %)			
Male	294,674 (41.7)	162,286 (43.2)	456,960 (42.2)
Female	409,133 (57.9)	212,866 (56.7)	621,999 (57.5)
Other/unknown	2774 (0.4)	411 (0.1)	3185 (0.3)

^a^Some cases (<0.1%) were missing age.

**Fig. 1:**
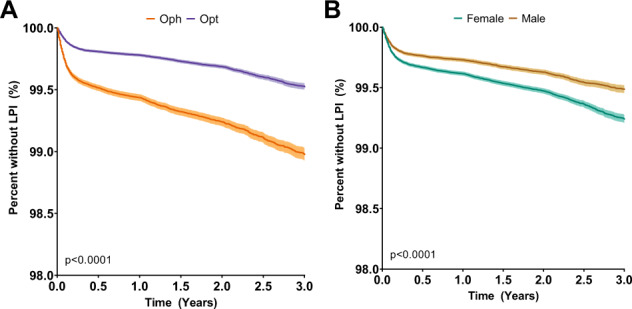
Kaplan–Meier curves on narrow angle detection in patients grouped by type of provider (a) and sex (b). *X*-axis: years since the first eye evaluation. *Y*-axis: percent of study population who has not received laser peripheral iridotomy (LPI). **a** Purple: patients seen by optometrists; Orange: patients seen by ophthalmologists; **b** Yellow: male; Green: female.

**Fig. 2:**
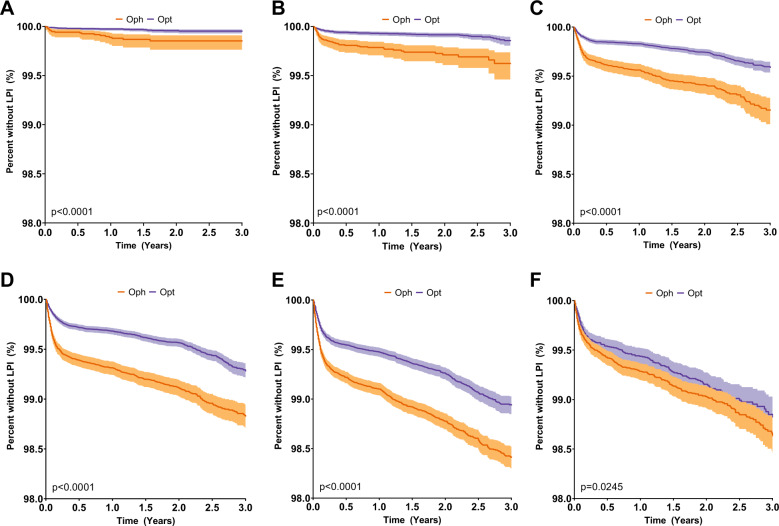
Kaplan-Meier curves on narrow angle detection in patients seen by either optometrists (purple) or ophthalmologists (orange), stratified by decades of life. *X*-axis: years since the first eye evaluation. *Y*-axis: percent of study population who has not received laser peripheral iridotomy (LPI). **a** age group 20–29; **b** 30–39; **c** 40–49; **d** 50-59; **e** 60–69; **f** 70+.

The rate of occludable angle detection was noted to be higher in females and per increase in decade of life (Figs. 1b and 2). Overall the detection of occludable angle was noted to be statistically significantly higher in the patients seen initially by ophthalmologists. Since the male vs female distribution between optometrists and ophthalmologists were similar, the Kaplan–Meier curves were stratified by decade of life to compare the rate of occludable angle detection by ophthalmologists and optometrists (Fig. 2).

The rate of occludable narrow angle detection was significantly higher in patients with older age, female sex, and those evaluated by ophthalmologists. The rate of LPI was significantly higher during the first 4 months after the initial assessment by either provider, therefore we accounted for the time interaction in our model (Table 2). On the Cox regression model that controlled for age category, sex and time × provider interaction, the hazard rate of occludable narrow angle detection was 46% lower in patients seen initially by optometrists compared to those who were seen initially by ophthalmologists. After controlling for provider type, sex, and time × provider interaction and using reference age category as 0–10 years, ages 11–20 had hazard ratio (HR) of narrow angle detection of 1.73 (*p* = 0.205), 21–30 HR of 4.5 (*p* = 0.0002), 31–40 HR of 10.47 (*p* < 0.0001), 41–50 HR of 27.9 (*p* < 0.0001), 51–60 HR of 46.12 (*p* < 0.0001), 61–70 HR of 68.37 (*p* < 0.0001), and 70–80 HR of 56.75 (*p* < 0.0001).

**Table 2:** Cox regression model for laser peripheral iridotomy as the outcome controlling for time × provider interaction effect.

Provider or age category	Hazard ratio	95% CI	*p* value
Ophthalmology	Reference group		
Optometry	0.54	0.50–0.59	<2e–16***
0–10	Reference group		
11–20	1.73	0.74–4.10	0.205
21–30	4.50	2.04–9.91	0.00019***
31–40	10.47	4.89–22.43	1.49e–09***
41–50	27.90	13.22–58.74	<2e–16***
51–60	46.12	21.94–96.96	<2e–16***
61–70	68.37	32.55–143.61	<2e–16***
70–80	56.75	26.94–119.56	<2e–16***
Female	Reference group		
Male	0.68	0.64–0.73	<2e–16***
Provider × time interaction	1.19	1.05–1.34	0.0047**

**p < 0.01; ***p < 0.001.

## Discussion

Our review of 1,082,144 new patients seen by an eye care provider in the state of MA during the years 2011–2015 reveals that narrow angles are associated with older age and female sex. In addition, the detection rate of narrow angles is significantly higher by ophthalmologists than by optometrists.

The true prevalence of narrow angles is difficult to assess without a population screening program, which would be costly and require trained personnel [9]. Instead, many have studied the prevalence or incidence of PACG which could be considered as a surrogate number for narrow angles. For example, one group performed a systematic review and modeled the prevalence of PACG in European countries and estimated that 1.6 million people in Europe and 581,000 people in the United States currently have PACG [14]. Although race information was not available in this database, the majority of the MA population is Caucasian and therefore the prevalence of PACG of our study population may be similar. Certain races (e.g., Eskimos, East Asians), female gender, and older age have been shown as important predictors of PACG, similar to what we found in our study [5, 9, 15]. We also found that the rate of narrow angle detection inferred by the rates of LPI was increasingly higher with older age (range 4.5–56.8) and males were 32% less likely to have narrow angle than females.

The higher detection rate of narrow angles by ophthalmologists compared to optometrists was unexpected. However, we performed several steps to minimize potential bias in our analyses. First, we only evaluated new patients who were seen by optometrists or ophthalmologists and excluded any follow-up patients. Second, since optometrists do not perform LPI, we included all LPIs that were performed by ophthalmologists within 90 days after the initial referral from optometrists as patients with narrow angles in the optometry group. Importantly, if patients were referred to ophthalmologists and no LPI was performed within 90 days, then patients were censored from the last date of evaluation with the optometrist. Third, we excluded any patients who received LPI on the same day patients were evaluated by eye care providers. The reasoning was that these participants received an emergent/urgent LPI likely due to symptoms and their narrow angles were not an incidental finding. These patients would have been more likely to present at ophthalmology offices rather than optometry offices. The fact that the rate of LPI was much higher in the first 4 months is not surprising and supports that narrow angles were detected and followed up with LPI as non-urgent cases in our population. These patients who received an LPI in the first four months most likely represent the latent undiagnosed population of patients who are asymptomatic and do not realize that they have narrow angles.

Lower rate of narrow angle detection in patients who are only followed by optometrists has important clinical implications. Undetected narrow angles increase the risks of incidence of acute angle closure (i.e., painful episode of acutely elevated intraocular pressure and vision loss) and PACG [16]. LPI can prevent the acute angle closure in a significant number of cases and patients can recover from angle closure without significant visual field defect if treated early [17]. Thus early detection with appropriate counseling in patients with narrow angles is critical for patients’ overall eye care, and eye care providers must be aware of the risks associated with narrow angles.

Both optometrists and ophthalmologists are critical components of eye care delivery in the USA, but these providers undergo substantially different types and durations of training. Optometrists undergo a 4-year optometry curriculum and there is no mandatory postgraduate training. Ophthalmologists are medical doctors who undergo 4-year of undergraduate education and 4-year medical school curriculum, followed by mandatory 1-year general medical or surgical internship and 3-year ophthalmology residency training. The Accreditation of Graduate Medical Education (ACGME) which regulates ophthalmology training requires that ophthalmologists manage a minimum of 3000 outpatient visits with a variety of ocular diseases [18]. In contrast, no accreditation criteria regarding the minimum requirements of patient visits exists for optometry schools [19]. While optometrists undergo more in-depth training in the management of non-surgical refractive error than ophthalmologists, ophthalmologists undergo more standardized and rigorous training in procedures such as gonioscopy or surgeries than optometrists [20]. These differences raise concerns regarding recently increased scope of practice for optometrists in some US states. In a review of Medicare patients who underwent a laser procedure for glaucoma treatment by either optometrists or ophthalmologists in Oklahoma, USA (one of the few states where optometrists have surgical privileges), the patients undergoing laser trabeculoplasty for glaucoma treatment by optometrists had nearly threefold higher hazard of requiring additional laser in the same eye compared to those receiving the same laser treatment by ophthalmologists (HR 2.89, 95% CI 2.00–4.17; *p* < 0.001) [21].

Limited literature exists comparing the glaucoma care including the ability to detect narrow angles between optometrists and ophthalmologists. Interestingly, a Scottish study published in the journal sponsored by the College of Optometrists revealed that only 59 of 95 patients who were referred to glaucoma clinic for suspected angle closure indeed had occludable narrow angles (62% positive predictive value [PPV]). In addition, 19 out of 620 patients that were referred to the glaucoma clinic for conditions other than narrow angles had narrow angles on gonioscopy performed by ophthalmologists (97% negative predictive value [NPV]). The authors concluded that community optometrists were effective in detecting eyes at risk of angle closure. The national eye care delivery model in the United Kingdom is significantly more integrated between optometrists and ophthalmologists than the USA [22–24]. The 62% PPV and 97% NPV of narrow angle detection by optometrists may be sufficient when most of the patients with questionable findings are referred to ophthalmologists but not when the referral to ophthalmologists is highly variable. A recent study published in an optometry journal showed fair-to-moderate concordance in gonioscopy (52–60% of the angle configurations were graded identically) between optometrists and ophthalmologists [25]. Unlike our results, however, the authors found that the agreement on the final diagnosis of angles being either open or closed was excellent.

We defined narrow angles as the ICD codes pertaining to narrow angles that led to LPI and the true prevalence of narrow angles is much higher than what we identified in this study. A recent single-center, randomized controlled ZAP trial from China showed a near 50% reduction (HR 0.53, 95% CI 0.30–0.92) in developing angle closure disease in 889 primary angle closure suspects who were randomized to LPI or observation [3]. During a follow-up period of 72 months, the incident angle closure disease occurred in a total of 19 treated eyes and 36 untreated eyes (*p* = 0.004). Notably, fewer than 1% of the study participants progressed to PACG, and the authors advised against widespread prophylactic LPI in resource-limited populations. Whether our study patients benefited from LPI or whether a different treatment should have been considered (e.g., phacoemulsification) [26] is beyond the scope of our study, and more longitudinal studies with different ethnic backgrounds are needed [27]. Of note, these studies were published more recently than our study period.

In the USA, both optometrists and ophthalmologists routinely screen for narrow angles, and the majority of these patients receive prophylactic LPI for narrow angles currently [16, 28]. Therefore, a correct diagnosis is essential so that patients can be counseled and followed appropriately. Given that the rate of narrow angle detection was substantially higher when patients were seen by ophthalmologists, evaluation by ophthalmologists may benefit patients who are at higher risk of PACG thus potentially decreasing the risk of complications related to undetected narrow angle. A recent study showed that 90% of the US Medicare population resides in close proximity of both ophthalmologists and optometrists, suggesting that the majority of our patients should be able to access ophthalmologists [29]. Nevertheless, in light of the recent ZAP trial results [3], the cost implications of population-based screening of narrow angles or prophylactic LPI especially in resource-limited settings should be evaluated in future studies.

Several limitations exist with our study. First, an important assumption of the study is that a similar population is being seen by optometrists and ophthalmologists in our dataset. Other unmeasured confounders may exist between the two groups that could explain the discrepancy in the rate of LPI. In our cohort, the patients seen by optometrists only tended to be younger, so we stratified all our analyses by each decade of age to better compare the two groups. Patients who were seen by ophthalmologists were older than those who were seen by optometrists and patients with several ophthalmic diseases were more likely to see an ophthalmologist than an optometrist. However, most other common ophthalmic conditions associated with age have not been associated with increased risk of narrow angles, which tend to be diagnosed incidentally. Second, we did not have data on laterality or visual outcome data. We were conservative and only assessed one eye per patient. More recently released MA APCD dataset that includes ICD-10 would provide laterality data for future studies. Third, we defined narrow angles in our study as the narrow angles that required LPI. The true incidence of narrow angles are likely higher given that the decision for LPI for occludable narrow angles can be somewhat subjective and vary between eye care providers. However, given the large number of our study population and eye care providers, we anticipated a similar LPI rate in patients seen by optometrists and those seen by ophthalmologists, but the rates differed significantly. Lastly, we did not have access to Medicaid populations in MA APCD and cannot evaluate whether a similar trend of LPI is found in low-income adults and children.

To summarize, in a review of 1.7 million person-year follow-up from over 1 million unique patients who received a new patient evaluation by either optometrists or ophthalmologists in the state of MA, we found older age, female sex, and evaluation by ophthalmologists to be associated with narrow angles. A baseline appointment with ophthalmology may benefit older patients, patients with complex ocular conditions that may affect the examination, or those who are at higher risk for PACG.

## Summary

### What was known before

- Eye care in the United States is currently provided by both optometrists and ophthalmologists.
- The training requirements and expertise of these groups differ significantly.
- Few studies have directly compared the quality of care between these provider types.

### What this study adds

- On a review of over 1.7 million person-year data of all healthcare claims from the state of Massachusetts, we examined the difference in the routine detection of narrow angles on standard eye examinations.
- Ophthalmologists detected a statistically significantly higher rate of narrow angles in both new and established patients compared to optometrists.
